# A novel versatile hybrid infusion-multielectrode recording (HIME) system for acute drug delivery and multisite acquisition of neuronal activity in freely moving mice

**DOI:** 10.3389/fnins.2015.00425

**Published:** 2015-11-05

**Authors:** Oleg Senkov, Andrey Mironov, Alexander Dityatev

**Affiliations:** ^1^Molecular Neuroplasticity Group, Deutsches Zentrum für Neurodegenerative ErkrankungenMagdeburg, Germany; ^2^Neurotechnologies Department, Institute of Biology and Biomedicine, Lobachevsky State University of Nizhny NovgorodNizhny Novgorod, Russia; ^3^Nizhny Novgorod State Medical AcademyNizhny Novgorod, Russia; ^4^Medizinische Fakultät, Otto-von-Güricke-Universität MagdeburgMagdeburg, Germany

**Keywords:** EEG, electrophysiology, oscillations, spikes, brain, hippocampus, infusion, pharmacology

## Abstract

To characterize information transfer in defined brain circuits involving multiple brain regions and to evaluate underlying molecular mechanisms and their dysregulation in major brain diseases, a simple and reliable system is ultimately required for electrophysiological recording of local field potentials (LFPs, or local EEG) in combination with local delivery of drugs, enzymes and gene expression-controlling viruses near the place of recording. Here we provide a new design of a versatile reusable hybrid infusion-recording (HIME) system which can be utilized in freely moving mice performing cognitive tasks. The HIME system allows monitoring neuronal activity in multiple layers in several brain structures. Here, we provide examples of bilateral injection and recordings of full spectrum of learning and memory related oscillations, i.e., theta (4–12 Hz), gamma (40–100) and ripple activity (130–150 Hz), in five hippocampal layers as well as in the CA1 and CA2 regions. Furthermore, the system is designed to be used for parallel recordings in the amygdala, cortex and other brain areas, before and after infusion of reagents of interest, either in or off a cognitive test. We anticipate that the HIME system can be particularly convenient to advance functional neuroglycobiological studies and molecular deciphering of mechanisms governing long-term memory consolidation.

## Introduction

There is a surge need for a simple, cheap, reliable, miniature, flexible, scalable, and versatile electrophysiological recording system which would allow for simultaneous multisite local field potentials (LFPs) recordings, possibly also unit recordings, and injection of drugs, enzymes and viruses for gene expression manipulations in the brain. In majority of methodological papers and protocols either electrodes (Korshunov, 1995; Lin et al., 2006; Jeffrey et al., 2013) or injecting cannulas (Kehr et al., 2001; Senkov et al., 2006; Kochlamazashvili et al., 2010; Mohammadi et al., 2015) are demonstrated to be efficiently used. Local delivery systems provided great insights into mechanisms of memory formation as they allowed injection of drugs, proteins and glycans at specific stages of memory acquisition, consolidation and re-consolidation (Senkov et al., 2006; Gogolla et al., 2009; Kochlamazashvili et al., 2010; Romberg et al., 2013; Happel et al., 2014).

Multisite recordings of brain oscillations gain valuable information about various aspects of cognition, perception and learning and memory in humans and animals (Amaral et al., 2006; Buzsáki, 2011). For instance, it was shown that theta and gamma oscillations, their synchrony and cross-coupling are particularly important for encoding memory traces in the hippocampus in different learning paradigms (Tort et al., 2009; Benchenane et al., 2010; Korotkova et al., 2010; Shirvalkar et al., 2010; Bott et al., 2015). Interestingly, slow and fast gamma oscillations in CA1 differentially rout information from CA3 and medial entorhinal cortex, respectively (Colgin et al., 2009), while hippocampal ripples are involved in cortico-hippocampal consolidation of memory (Nokia et al., 2012), and beta frequency (20–40 Hz) is involved in coordination of entorhinal-hippocampal ensemble activity during associative learning (Igarashi et al., 2014). Multisite LFP recordings in the cortex revealed the role of gamma-oscillation coupling in binding of sensory information (Minlebaev et al., 2011), while parallel recordings in the prefrontal cortex (PFC), amygdala and hippocampus highlighted correlates of fear learning (Seidenbecher et al., 2003; Narayanan et al., 2007; Lesting et al., 2013) and a coherent coupling of low theta (4 Hz) and gamma oscillations upon working memory load in PFC, ventral tegmental area (VTA) and hippocampus (Fujisawa and Buzsáki, 2011).

Here, we propose a system which combines intracranial injection guide cannulas and multiple bundles of electrodes to enable long-time recordings (2–3 months) in multiple brain locations/multiple hippocampal/cortical layers before and after local application of a reagent of interest in small rodents, like mice, performing a complex, and sensitive to animal discomfort cognitive tasks (e.g., novel object recognition test, NORT). We believe that this technology will open new possibilities for mechanistic studies and advance further development of methods of this art in the neuroscience field.

## Materials and methods

### Animals

Adult (2–3 month old) male C57Bl/6j mice (Charles River, US) were used. At least 1 week before starting the experiments, the mice were transferred from the major animal facility of DZNE (Magdeburg, Germany) to a small vivarium, where they were housed individually with food and water *ad libitum* on a reversed 9:9 light/dark cycle (light on at 9:00 p.m.). All behavioral experiments were performed at the afternoons during the dark phase of the cycle when mice are active, under constant temperature (22 ± 1°C) and humidity (55 ± 5%). All treatments and behavioral procedures were conducted in accordance with ethical animal research standards defined by German law and approved by the Ethical Committee on Animal Health and Care of the State of Saxony-Anhalt, Germany, the license number: 42502-2-1159DZNE.

### Fabrication of electrodes, cannulas and headstages

#### Electrodes

We have designed new electrodes which are easy and fast to build, handle and implant. Each of 2, 3 or 5 electrode bundles/arrays were made of tungsten wires (50/112 μm) insulated with Teflon (originally from A-M Systems, USA, distributed in Europe by Science Products (SP), Hofheim, Germany) bound together in a stainless steel tubing (0.7 mm, 0.5 mm, or 0.4 mm, depending on number of wires inside, see Table 1), and isolated from it by plastic polyimide tubing (0.5 mm, 0.25 mm, 0.16 mm Neuralynx, and SP see Figures 1B, 3C). Fabrication of a single 5-electrodes-array takes about 10–15 min. First, the stainless tube (0.48/0.71 mm) was cut by an electrical miniature circular saw (“Dremel” Work Station, Model 8200) to the length of 8 mm and then its edges were polished under 90° angle by using the same machine with abrasive circular cutting wheels. After this procedure, the final length of the tube became 7 mm. The opening of the tube was cleaned by an insect pin (size 2, 0.45 mm) and compressed air flow (Air Duster, Servisol), and then, inside of this tube, another plastic tube (0.34/0.5 mm, Neuralynx, USA, length 8 mm) was inserted in a way that one of its endings was beyond the metal tube by 1 mm. Next step in fabrication was to insert wire electrodes (cut in proper length beforehand) inside the plastic tube and to arrange tips of each electrode accordingly to the mouse brain atlas (Franklin and Paxinos, 2007) coordinates to aim electrodes to different locations, e.g., to different hippocampal layers. For the latter, we used a decrement of 250 μm between each electrode, starting from 2 mm length, to reach *molecular layer* of the dentate gyrus (DG), and ending with the shortest electrode, 1–1.1 mm, to record from *stratum oriens*. To know exactly which wire tail (must be connected with EIB-16 later) coming out the electrode corresponds to which electrode (see Figure 1B), we used “a length code”: deeper an electrode/wire is arranged in the electrode array, shorter it has a tail, so the most superficially located electrode must have longest tail (see Figure 1B). The final step was to fix the electrodes with proper lengths and arrangement of tips inside of the metal-plastic tube by gently compressing it with a small (300 g) hammer, hitting it from both sides of the metal tube by a small chisel. Such mechanical fixation rather than fixation with an epoxy glue (we tried to use), with 100% warranty ensures absence of any misplacements of electrodes, even when their tails are manipulated during pinning/unpinning to/from the EIB-16.

**Table 1:** **Construction details for electrodes**.

Number of Teflon insulated Tungsten wires (Ø50/112 μm)^*^	**Polyimide tubing size OD**	**Stainless steel tubing size ID/OD**
1	Ø127 μm^*^	Ø165/330 μm^*^
2	Ø250 μm^*^	Ø292/508 μm^*^
3	Ø250 μm^*^	Ø292/508 μm^*^
4	Ø497 μm^#^	Ø483/711 μm^*^
5	Ø497 μm^#^	Ø483/711 μm^*^

* Science Products/A-M Systems;

# *Neuralynx*.

**Figure 1:**
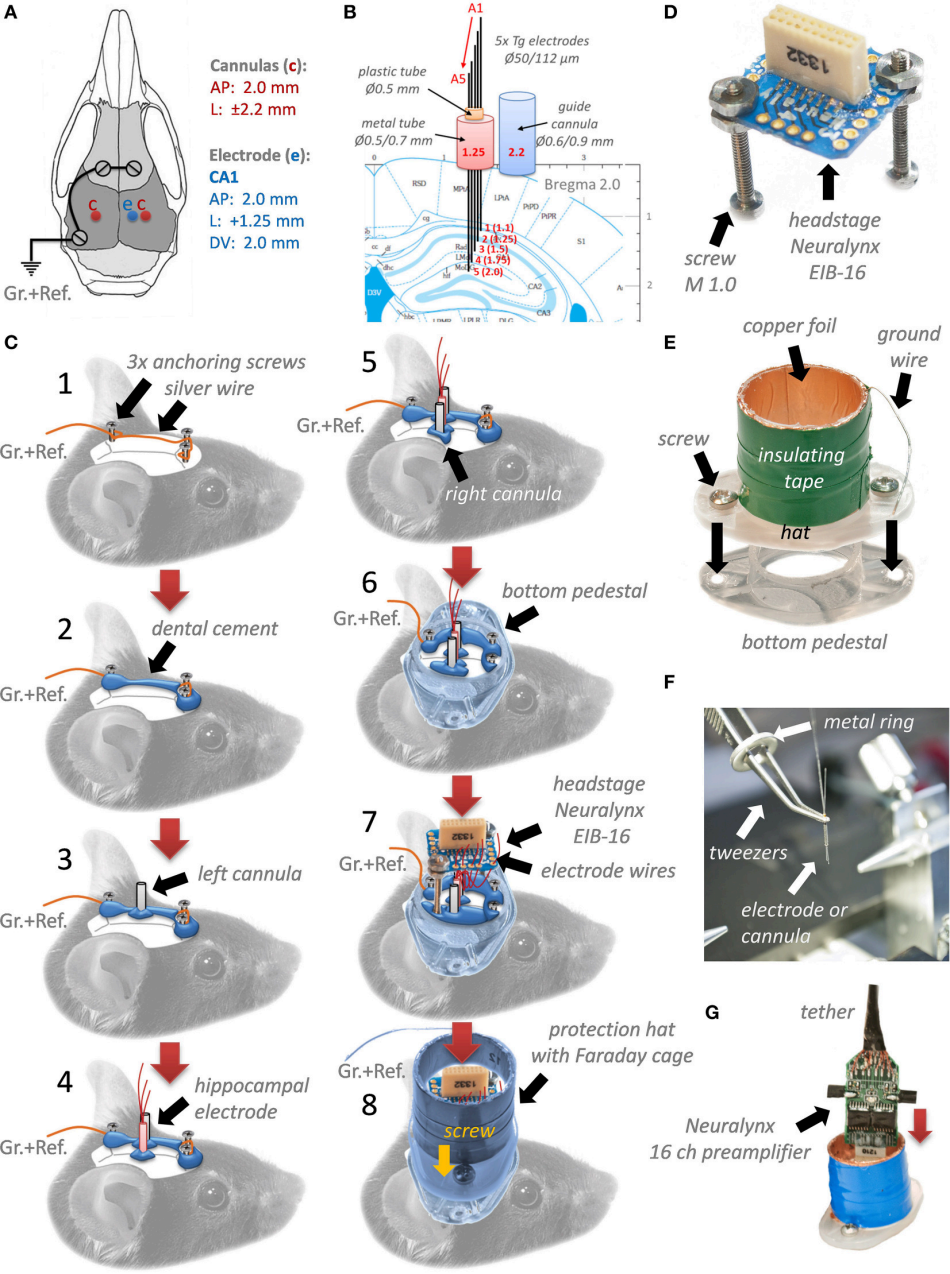
**Surgery procedure of implantation of hippocampal electrodes and bilateral injection guide cannulas in the mouse brain**. Coordinates of implantation and position of ground (Gr.) and reference (Ref.) electrodes **(A)**. A scheme of implanted hippocampal multilayer electrode array and guide cannulas **(B)**. Surgery steps **(C)**. Neuralynx electrode-interface board (EIB-16) for 16 channels **(D)**. Construction of a protective mouse hat and a Faraday cage around electrodes, cannulas and EIB-16 **(E)**. The way how to handle and implant electrodes and cannulas by using tweezers and a metal ring **(F)**. Implantation setup connected to a preamplifier and a tether **(G)**.

#### Cannulas

To reduce the production costs of the designed system, we decided not to use commercially available guide cannulas, but instead to make them ourselves. We used the same stainless steel tubing from SP as for electrode fabrication. The tube was cut to a proper length (9 mm) by the same saw machine as used for electrode fabrication. The fine polishing reduces final length of the tube to 8 mm. To fabricate dummy cannulas, stainless steel insect pins or push pins for cork notice boards can be used. Since the inside diameter of the guide cannula is 0.64 mm (OD 0.90 mm), we used pins with 0.60 mm so they as tight as possible fitted to the guide cannulas. The pin was cut first to 11–12 mm, then, one of its ends (2–3 mm) was curved by 90° with pliers to make a small hook, which could later be used to pull out dummy cannulas from guide cannulas before injection. To make perfect match in lengths of dummy and guide cannulas, the dummy cannula was fully inserted into the guide one and its excessive length was removed by polishing with the circular abrasive saw “Dremel.” If required, e.g., to match closer cannulas and electrodes, the size of guide cannulas can be decreased to 0.71/0.48 mm, since the shank of the injecting “NanoFil” needle is 0.43 mm. Before implantation, all cannulas were cleaned with 75% ethanol, dried and oiled with a sterile mineral oil (Sigma Aldrich, Germany) to prevent clutching of the guide cannula by a dummy cannula after implantation due to capillary forces which might otherwise attract salty fluids over the time after craniotomy.

### Implantation of electrodes and cannulas

#### Surgery

Chronic implantation of electrodes and cannulas in mice was performed similar as described previously (Senkov et al., 2006; Kochlamazashvili et al., 2011) with minor changes. In brief, mice were anesthetized with a 1–3% *Isoflurane* (Baxter International Inc.) delivered as a mixture with O_2_ through a Vaporizer (Matrx VIP 3000, Midmark, Versailles, USA, www.midmark.com) and a mouse breathing mask. Prior to any surgical manipulation, the mouse was given the analgesic *Carpofen* (5 mg/kg b.w. s.c., Rimadyl, Pfizer Pharma GmbH), its head hairs were shaved by using a small clipper (Contura, HS61, Wella), its skin was cleaned by *75% Ethanol*, followed by 10% *Povidone Iodine* (Dynarex, Orangeburg, Mexico) as a topical long-lasting antiseptic, then an additional analgesic *Xylocain* was used on skin (a pumpspray, 10 mg *Lidocain*, Astra Zeneca GmbH, Wedel, Germany).

The mouse then was placed in a stereotaxic frame (Narishige, Japan), and all next procedures were performed under a surgical binocular microscope (Labomed Prima DNT, Labo America Inc., Fremont, USA, www.laboamerica.com) and on a heating pad (DC Temperature Controller, WPI) to maintain mouse body temperature constant (34–36°C) over surgery. The mouse scalp skin was circular incised (10 mm) and removed (see Figure 1C1). The edges of the skin were processed with *75% Ethanol* and *Xylocain*. The scalp bone was carefully cleaned from tissue by using *75% Ethanol* and *3% Hydrogen Peroxide*, H_2_O_2_, then, dried with a heating drier at the lowest temperature (< 40°C) and speed (Steinel GmbH, HG-2310 LCD, Herzebrock-Clarholz, Romania). Treated with H_2_O_2_ bone better reveals position of *bregma, lambda*, sutures, and big capillaries under the bone. This helps to correctly position electrodes and cannulas in the brain, avoiding excessive bleeding. After marking coordinates for implantation, 3 small holes for anchoring and ground screws (see Figure 1C1) were drilled in two frontal bones and one in the left parietal at the border with the interparietal bone by using a dental micro motor (Eickemeyer, Tuttlingen, Germany, www.eickemeyer.de). Then in frontal holes, two small screws (Plastic One) and in the parietal hole a screw with soldered ground/reference wire were gently inserted (see Figure 1C1). Two frontal screws were connected with silver wire with the parietal ground screw (see Figure 1C1). Then all three screws and wire were covered with acrylic dental cement Paladur (Heraeus Kulzer GmbH, Hanau, Germany) leaving the marked areas for electrodes and cannulas free of acrylic (see Figure 1C2). Implantation began with a drilling of a hole for the left cannula (see Figure 1C3). The cannula was stereotaxically implanted by using a self-made universal holder for cannulas and electrodes (a small curved tweezers from FST with a metal ring, see Figure 1F). During implantation the cannula gently touched the surface of the brain and only then it was secured with a small amount of acrylic. Next, the hippocampal electrodes were implanted in the right parietal bone (see Figure 1C4). The procedure was similar to implantation of the cannula. During implantation the deepest hippocampal electrode was advanced until it gently touched the surface of the brain, setting the starting point (“0”) for inserting the whole electrode bundle (2 mm beneath of the brain surface). After hippocampal electrodes, the right cannula is similarly implanted and secured with acrylic (Figure 1C5). Coordinates for bilateral cannulas (AP: −2.0 mm; L: ±2.2 mm from Bregma, Figure 1A; bottom end of cannulas should touch the surface of the brain), and for the bundle with 5 electrodes reaching different layers of the hippocampus (AP: −2.0 mm; L: ±1.25 mm from Bregma; DV: 2.0 mm from the brain surface, Figure 1A) were set according to the mouse brain atlas (Franklin and Paxinos, 2007). After implantation of cannulas and electrodes, more dental cement was used to secure the whole system on the bone. Next step was to glue a bottom part of the pedestal (using dental cement) to the skull, positioning it optimally for the mouse and implants, and as horizontally as possible (Figure 1C6). After the whole system is well secured, the Neuralynx EIB-16 (Figure 1D) headstage with two screws was positioned on the top (Figure 1C7) and glued with acrylic over two screws. Once this was done, all the Tungsten wire tails from electrodes, starting from the longest tail (corresponding to the most superficially located electrode) were gently inserted into channels holes on the EIB-16, and pinned by golden pins (Neuralynx). The pins provide mechanical connections between electrode wires and the EIB-16 channel inputs. Removal of Teflon insulation from wires was not necessary; golden pins perfectly do it themselves during insertion. After assembly was completed, all ground wires were soldered together and the upper cover “hat” was placed above the bottom pedestal and fixed to it with two small screws (Figures 1C8,E). The protecting “hat” with a Faraday cage was made from a standard 10 ml syringe (17.4 mm) by cutting off its “ears” part by 15 mm (see Figure 1E), then wrapping it with one-side-self-adhesive copper foil soldered with silver wire for grounding, and covered for protection with another layer of plastic self-adhesive tape. The bottom pedestal was made of another 5 ml standard syringe (14 mm), which perfectly fitted inside 10 ml syringe used for hat fabrication, by cutting its “ears” part off by 2 mm. The “hat” could be fixed on the bottom pedestal by using two small screws (see Figure 1E). To cover the top opening of the “hat,” we used a black rubber plunger tip from 10 ml syringe that was used for the hat: it perfectly fits and easy to put in/out, if one cuts a small ~10% piece of it. After the surgery lasting for 90 min, mice were placed back into their home cages and monitored until full awakening, which usually lasted about 15–20 min. *Carpofen* (5 mg/kg b.w. s.c., Rimadyl, Pfizer Pharma GmbH) was used as a postoperative analgesic. All behavioral investigations and recordings were performed after the mice had fully recovered 5–7 d after surgery. Recordings were done by using Neuralynx 16 channels preamplifier and 5 meters tether (Figure 1G).

### Intrahippocampal injection

For intrahippocampal injection we used a digitally controlled infusion system (UltraMicroPump, UMP3, and Micro4 Controller, WPI, www.wpiinc.com USA) fed with a 10 μl Hamilton syringe and NanoFil 135 μm (35 GA) beveled needle. The thin (135 μm) part of the needle went into the mouse brain during injection, whereas its thick part (430 μm) perfectly fitted to the guide cannula (inside 600 μm). Injections were done in the following manner: (1) a mouse was anesthetized with a 1–3% *Isoflurane* as during implantation; (2) it was put into the stereotaxic frame; (3) the headstage EIB-16 was gently unscrewed and shifted a bit away to expose underneath electrodes and cannulas. Since electrodes were pinned to EIB-16 by having rather long 20–25 mm length tails, EIB-16 could be easily and safely shifted aside without disconnecting electrodes; (4) the dummy cannula was gently removed from the left guide cannula; (5) NanoFil injecting needle was accurately placed inside of the guide cannula and advanced further down till the marker (marker was put with a permanent pen at the needle shank (430 μm) to visualize the point when the needle should reach the surface of the brain coming out of the guide cannula (equal of the length of guide cannula); (6) once the marker was reached, a slow 10 μm increment step dial is used to deepen the needle into the brain; (7) injection was done at the brain area of interest; for example, we injected MK801 (0.5 μg/0.5 μl per site, Sigma -Aldrich, Germany) or vehicle as deep as 2.0 mm from the brain surface (injection rate was 3 nl/s); (8) after injection was complete (~3 min), the needle was left in the place of injection for other 5 min, and then gently removed; (9) the dummy cannula was then placed back; (10) the same procedure was done for the right hemisphere; (11) then EIB-16 was returned to its place and screwed back. The whole injection takes approximately 20–25 min under *Isoflurane* anesthesia, and the mouse recovers rather fast, in 2–3 min it started to move in its home cage.

### Behavior

#### Novel object recognition test (NORT)

To validate that our HIME system is suitable for cognitive experiments, we performed rather sensitive to animals discomfort novel object recognition task at day 1 (d1) and day 14 (d14) after intrahippocampal injection of a vehicle (see **Figure 3E**). Each day every mouse went through 2 trials of NORT separated by 1–2 h. Each trial consisted of an encoding phase and a testing phase in an open field (OF) with the size of 50 × 50 × 30 cm. During the encoding phase a mouse was exploring two identical objects for 10 min, while in the testing phase (10 min) one already familiar object was replaced with a new one. The interval between encoding and testing phases was 1 h. In each trial we used different sets of objects. Spatial position (left or right) of the novel and familiar objects in OF was counterbalanced. The behavior of the mice during NORT was recorded on a HP workstation Z400, with Xeon computer (CPU 3.07 GHz, 6 GB RAM) using an USB video camera (Microsoft, USA) and a special behavioral video acquisition and analysis software (AnyMaze, USA). All recorded movies were then analyzed using AnyMaze by a trained observer blind to the type of the object, novel vs. familiar. Two trials per day were averaged and presented as a mean ±SEM.

### Statistical analysis

To analyze cognitive ability of tested mice to discriminate between familiar and novel objects, we used two-way ANOVA (SigmaPlot, ver 12.5, www.sigmaplot.com, Systat Software, San Jose, USA) with two factors: the day and the type of object, familiar, or novel; where significant differences were found, the *post hoc* Fisher LSD test was applied.

### LFP recordings

Intrahippocampal local field potentials (LFPs) were recorded by using a state-of-art digital electrophysiological 64 channels recording system (Neuralynx, USA) and with its original electrophysiological data acquisition software Cheetah (Neuralynx, USA). The raw signal was split for recording simultaneously LFPs and single units. LFPs were recorded at 3200 Hz sampling rate, 0.1–300 Hz wide-band filter, x1000 amplification, whereas units were sampled at 32 KHz at wide-band 0.1–10 kHz range filter, and stored at the local network server. LFPs were analyzed by using electrophysiological data analysis software package NeuroExplorer, ver 5.0 (www.neuroexplorer.com, Madison, USA). To be able to record a mouse in rather big open field (50 × 50 cm) we used a long (5 m) tether (Neuralynx) counterbalanced with an equal weight (20–30 g) of the headstage and the cable itself via a simple 2x rolling blocks system, that the mouse can freely move without any discomfort (see **Figure 3B**). To prevent twisting the tether cable over 10 min of recording, the cable must be at least 2 m long from the point where it is connected to the mouse to the point at the ceiling where it counterbalanced with the 2x blocks system (see **Figure 3B**).

## Results

**Figure 2:**
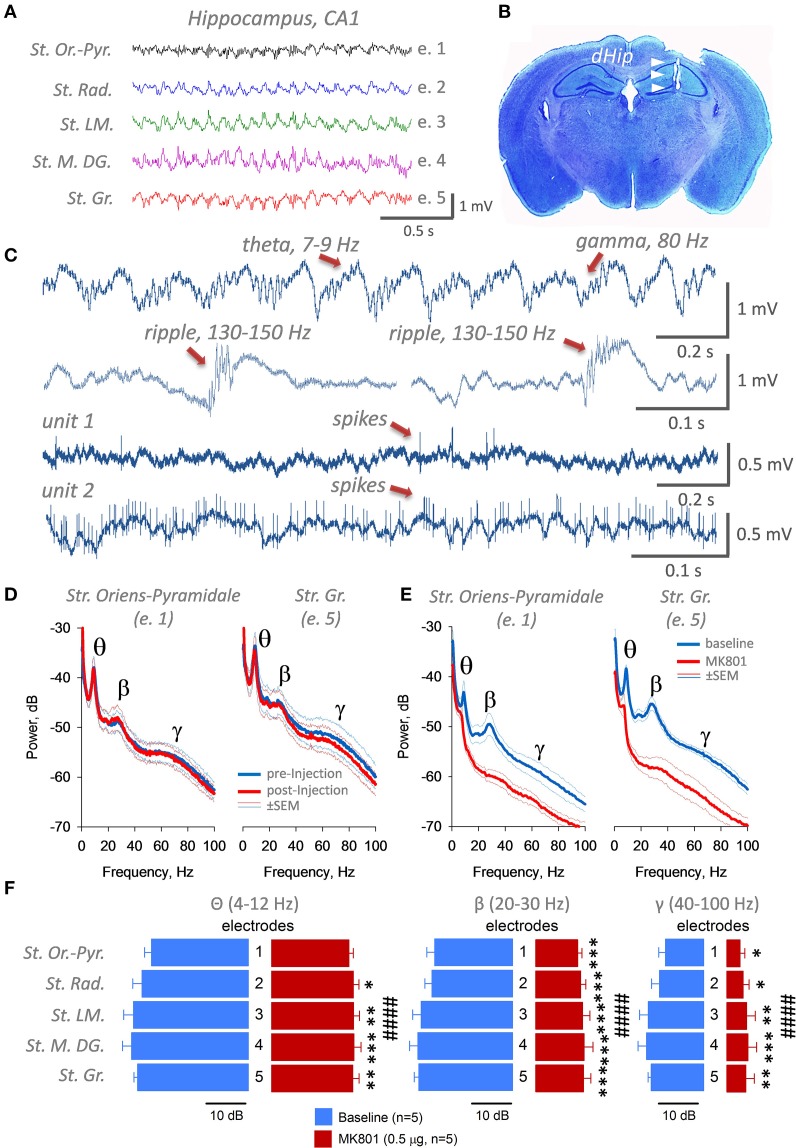
**Quality of LFPs (A,C) and unit (C) recordings with the multielectrode (e.1–e.5) array targeting 5 different layers of the hippocampus**. St. Or.-Pyr. *Stratum Oriens Pyramidale, St. Rad. Stratum Radiatum, St. LM. Stratum Lacunosum-Moleculare, St. M.DG. Stratum Moleculare of Dentate Gyrus, St. Gr. Stratum Granulare*. **(B)** An example of histological verification of electrode position in the dorsal right hippocampus, Nissl staining. **(D)** Verification of quality and power of oscillations after injection of a vehicle by analysis of signals at the first and fifth electrodes after the injection. Intrahippocampal bilateral injection of a vehicle did not influence power and frequency of θ-, β-, and γ-oscillations. **(E)** Power spectra density (dB) of hippocampal oscillations recorded before and after MK801 (0.5 μg/site) intrahippocampal injection. **(F)** Normalized power spectra densities and statistical evaluation for different frequency bands and hippocampal layers after MK801 injection. ^####^*p* < 0.001, Two-way ANOVA with *post hoc* Fisher LSD, *p < 0.05, **p < 0.01, ***p < 0.005.

After fabrication of all components (1 days) and implantation of 5 mice (2 days) with 2 intrahippocampal cannulas and one 5-electrodes array each, we performed electrophysiological recordings in their home cages for a few next days to check quality of LFP and single unit signals (see Figures 2A,C). If one of the channels did not properly receive the signal, repining of the wire on the EIB-16 usually helped to troubleshoot the problem. The hippocampal multielectrode array was designed in a way to record from different layers of the right dorsal hippocampus (see Figures 2A,B). This allows to collect information about different synaptic inputs and outputs of the hippocampus, e.g., the electrodes at *st. oriens* would pick up an output LFP signal coming from CA1 to the dorsal subiculum and EC (layer V), electrodes at *st. pyramidale* might catch spiking and ripple activity, electrodes at *st.radiatum* would register information flow coming from CA2/CA3 areas, electrodes at CA1 *st. lacumosum-moleculare* can rout input from EC (layer III) and electrodes at DG *st. moleculare* will “listen” to a major gateway input conveyed multimodal sensory and spatial information to the hippocampus from EC (layer II) through a perforant path.

In our case, tungsten electrodes (50/112 μm) could pick up prominent EEG signal in theta (4–12 Hz), betta (20–30 Hz), gamma (40–100 Hz), and ripples (130–150 Hz) ranges, as well as quite often single unit or multiunit spiking activity nearby *stratum pyramidale* (Figure 2C). Usually, LFP signal was very clean and free of artifacts and noise. Here, we give two examples of units recorded from a close proximity to *stratum pyramidale*: unit 1 is presumably one principal excitatory cell, whereas, unit 2, is probably one of a few interneurons. In general, more electrodes are implanted, the greater chances are to pick up unit activity with such not movable electrodes. In our experiments, almost every mouse we recorded had 1–2 channels with spiking activity, which could last usually for 5–7 days. To verify first that injection of a vehicle does not cause any change in oscillatory activity in the hippocampus, we recorded 5 mice at day 1 for 10 min in a home cage, then at day 2 we injected them with a vehicle, and then performed again LFPs recordings at day 3. As shown at Figure 2D, averaged power spectra densities as well as major peaks of oscillations (θ, β, and γ) did not significantly change after intrahippocampal injections with the vehicle. To validate that we can manipulate oscillatory activity in the dorsal hippocampus *in vivo*, we decided to inject an uncompetitive NMDARs blocker MK801 (0.5 μg per site), as it is known that it can impair different types of memory injected intrahippocampally in rodents (Ohno and Watanabe, 1995; Huang et al., 2004). We recorded LFPs in 5 mice for 10 min before and 10 min after of MK801 injection (~20 min). Power spectra density was computed and analyzed *post hoc*. As it seen at Figure 2E, normalized power (dB, 1st electrode and last one) of oscillations is significantly reduced after MK801 in θ, β, and γ ranges in all 5 hippocampal layers (Figure 2F). Oscillatory activity in theta/gamma range in the hippocampus is based on excitatory granule-pyramidal-pyramidal trisynaptic loop circuitry, as well as on excitatory drive into inhibitory GABAergic interneurons. Blocking with MK801 active synapses expressing NMDA receptors should decrease excitatory drive on all elements of the trisynaptic circuitry, and thus might reduce power/amplitude of theta/gamma oscillations. Two-way ANOVA with Fisher LSD showed major effect of the drug on all three bands of oscillations (*p* < 0.001) and it was independent of the hippocampal layer (*p* = 0.8).

**Figure 3:**
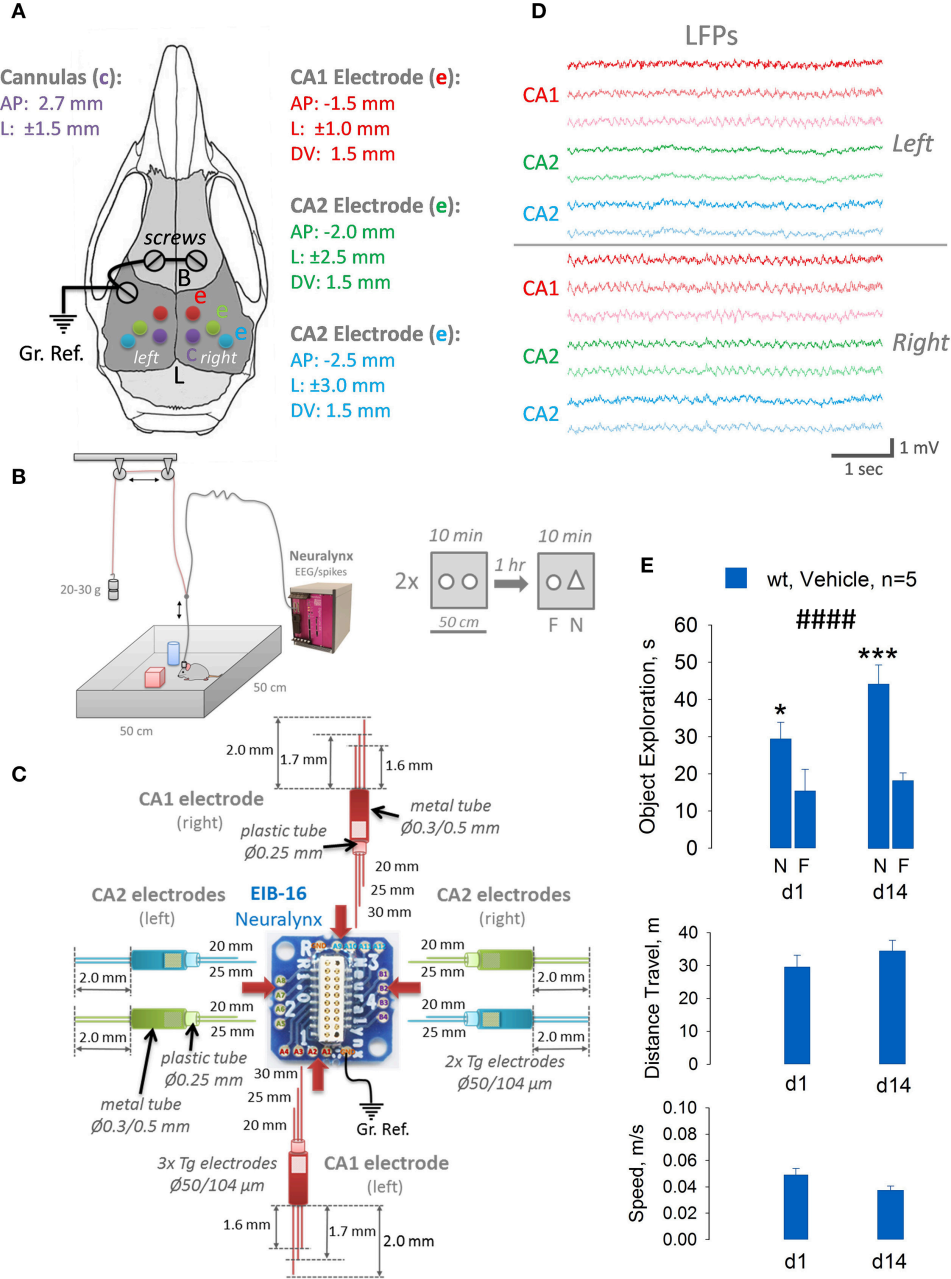
**Design and construction of the CA1/CA2 hippocampal multielectrode arrays of different complexity (C), implantation coordinates for cannulas and electrodes (A), quality of recording from 6 brain areas simultaneously (D) and behavior evaluation of implanted and injected with a vehicle mice in a sensitive novel object recognition task (B,E)**. Obviously, animals explore a novel (N) object more than a familiar (F) one on day 1 (d1) and day 14 (d14) with standard novel object exploration time for this test (30–40 s during 10 min in OF), distance traveled (~30–40 m) and running speed (0.04 m/s). ^####^*p* < 0.001, Two-way ANOVA with *post hoc* Fisher LSD, *p < 0.05, ***p < 0.005.

To validate that our technology can be easily used for multisite recordings from several brain areas simultaneously, together with a possibility to inject locally drugs of interest *in vivo* before cognitive tasks, we decided to implant five mice, each with six electrodes and two cannulas (Figure 3A). We chose to implant two 3-wires electrodes into CA1 area of the dorsal hippocampus (left and right, see Figures 3A,C), and four 2-wires electrodes into CA2 areas in both hemispheres. It took us about 2–3 days to fabricate all necessary electrodes and cannulas and a week to implant them. After recovery and quality control of LFP signals (Figure 3D) in all electrodes, mice underwent training in a novel object recognition task (NORT) for 1–2 weeks. As we can conclude from recordings in 5 animals, theta oscillations from CA1 area had a tendency to be more powerful and synchronous compared to CA2 area, and in the right hemisphere compared to the left one. But these data still need to be verified in additional cohort of mice and will be presented in details elsewhere. Once mice reached significant discrimination level between novel and familiar objects, we intrahippocampally injected them with a vehicle and tested next day (d1) and 2 weeks afterwards (d14) by using NORT. As it shown at Figure 3E, after injection, mice had normal activity in the open field, distance traveled was around 30 m for 10 min and average speed of 0.04 m/s. Two-way ANOVA with Fisher LSD showed significant levels of object discrimination (*p* < 0.001), which were not significantly different between day 1 and day 14 (*p* = 0.23). Thus, we can conclude that implantation of a rather complex multisite electrode/cannulas setup, additionally, intrabrain injection of the vehicle and tethering the mouse with a cable during a very sensitive to any distortions cognitive task as NORT in order to record local EEG, is technically and behaviorally possible, and can yield trustworthy data.

## Discussion

Here, we propose a new approach how to design and implant a hybrid infusion-multielectrode recording system, which we named “HIME” (in Japanse, 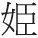 stands for “princess”) to be able to perform high quality electrophysiological recordings in freely moving mice after intracranial injection of a drug of interest before a behavior task. The system appeared to be quite simple, reliable, scalable, flexible, and versatile. Its parameters are given in Table 2. Majority of the components of the system can be recycled and reused many times, e.g., cannulas, EIB-16, pins, screws, hats, and bottom pedestals. It was also easy to fix problems with quality of EEG signals via repining loose wire electrodes, and/or re-soldering a ground electrode, since a protecting “hat” with the Faraday cage can be quickly removed, exposing well all connections between the headstage, electrodes, and cannulas. Because of all these innovations, the quality of signals is very good, with almost no movement, static or AC artifacts, which allowed us to record single and multiunit activity and ripples at electrodes next to *stratum pyramidale*. Here, for the first time to our knowledge, we show that by using our HIME technology, it is possible to implant electrodes of different complexity in multiple brain areas in combination with an injecting system which is located in close proximity (300–500 μm) of the local EEG-recorded circuitry. It is very easy to adapt our HIME system for recordings and injection of reagents in other brain areas than the hippocampus. It should be also possible to implant electrodes in remote from each other brain structures, like amygdala, mPFC, visual and auditory cortices, and infuse reagents of interest in one brain area, while recording in others. Studies with local EEG recordings addressing learning and memory mechanisms (Seidenbecher et al., 2003; Narayanan et al., 2007; Lesting et al., 2013) would strongly benefit by having at hand such system.

**Table 2:** **Parameters of the developed infusion-recording system**.

**Parameter**	**Values**
Number of injection cannulas	1–2
Number of recording electrodes	1–16
Number of electrode bundles	1–8
Signal amplitude^*^	≈1 mV
Noise level (peak-to-peak)^*^	≈50 μV
Time for single component production^#^	~10 min
Time for the whole HIME assembly^#^	< 1 h
Time for implantation of HIME^#^	< 90 min
Weight^#^	≈2 g
Cost^#^	< 100 Euro
Durability	>3 months

* with Tungsten 50 μm wire, baseline recordings in the CA1 str. pyramidale;

# *a reusable system with 2 injection cannula and one bundle of 5 electrodes*.

Previously, several other systems were developed for simultaneous injection and EEG/spikes recording at one brain structure. For instance, commercial solutions are provided by two companies (http://www.plastics1.com, www.neuranexus.com) with their “combo” electrodes and silicon neural probes for drug delivery, respectively. However, from our point of view, these systems would be too bulky to be used for multisite recording in mice, and they are rather “inflexible” in terms of implantation design of multiple electrodes, and costly. Another system was designed as cannulated microelectrode array for use in behaving rats, which enables neural ensemble recordings and local infusion of drugs in the same brain area (Hoffmann et al., 2011). However, the system is too big to be used for mice and requires fluid and electrodes swivels, so the animals are quite restrained and cannot be tested in large mazes, such as T-maze or a maze used for object recognition tasks. Another hybrid cannula-electrode device was developed for rats (Greger et al., 2007), but it is again too massive to be used for mice and it is rather for superficial cortical recordings and injection. Recently, an elegant approach was proposed for simultaneous optical, electrical and chemical interrogation of neural circuits *in vivo* (Canales et al., 2015), but this technique for fabrication of a multimodal optical fibers fused together with electrodes and mechanical parts is yet too complex and laborious, although quite advanced and precise. Several other groups produced microelectrode neural probes fused together with injecting capillaries (Rohatgi et al., 2009; Taylor et al., 2014; Lee et al., 2015; Shin et al., 2015), but they also require special machining tools and fabrication skills, multiple production stages, not recyclable parts, and thus they are yet very costly and cannot be easily replicated in a regular electrophysiological laboratory.

As injection in our system requires a short anesthesia (20–30 min), the injected compounds have to have long-term effects to allow recordings and behavioral analysis after post-anesthesia recovery. For instance, this is appropriate for experiments involving blockade of synaptic plasticity with NMDA receptor antagonist MK801, injection of extracellular proteases, such as matrix metalloproteinase MMP-9 (Wlodarczyk et al., 2011) or enzymes that digest particular glycans, such as endoneuraminidase for polysialic acid (Kochlamazashvili et al., 2010), chondroitinase ABC for chondroitin sulfates (Pizzorusso et al., 2006) and heparinase for heparan sulfates (Korotchenko et al., 2014). The functions of these and other carbohydrates carried by cell adhesion and extracellular matrix molecules have been mostly studied *in vitro* (Dityatev et al., 2006; Senkov et al., 2014), and we anticipate that our HIME system will facilitate translation of *in vitro* insights into mechanistic *in vivo* studies. Also the HIME system opens a new avenue for studies of systems for consolidation of long-term memories, which would benefit from simultaneous multielectrode recordings in the hippocampus and multiple cortical areas in combination with viral delivery in one of these areas to knockdown or gain expression of candidate genes responsible for memory transfer from the hippocampus to cortex (after initial acquisition of memories).

## Author contributions

OS: designed the method, performed the experiments, analyzed data, wrote manuscript and prepared figures; AM: designed the method, did surgeries; AD: wrote manuscript and prepared figures.

### Conflict of interest statement

The authors declare that the research was conducted in the absence of any commercial or financial relationships that could be construed as a potential conflict of interest.
